# Effects of forest structure on the endoparasitism in roe deer *Capreolus capreolus*

**DOI:** 10.1051/parasite/2025041

**Published:** 2025-07-25

**Authors:** Léa Bariod, Sonia Saïd, Hubert Ferté, Slimania Benabed, Hervé Bidault, Jeanne Duhayer, Sylvia Pardonnet, Gilles Bourgoin

**Affiliations:** 1 Université de Lyon, VetAgro Sup - Campus Vétérinaire de Lyon, Laboratoire de Parasitologie Vétérinaire 69280 Marcy-L’Etoile France; 2 Université de Lyon, Université Lyon 1, CNRS, UMR 5558, Laboratoire de Biométrie et Biologie Évolutive 69622 Villeurbanne France; 3 Office Français de la Biodiversité, Direction de la Recherche et de l’Appui Scientifique, Service « Conservation et Gestion des Espèces à Enjeux », « Montfort » 01330 Birieux France; 4 EA 7510, ESCAPE – Vecpar, UFR de Pharmacie, Université de Reims, Champagne-Ardenne 51100 Reims France; 5 Office Français de la Biodiversité, Direction de la Recherche et de l’Appui Scientifique, Service « Conservation et gestion durable des espèces exploitées » 79360 Villiers-en-Bois France; 6 Office Français de la Biodiversité, Direction de la Recherche et de l’Appui Scientifique, Service « Santé de la faune et fonctionnement des écosystèmes agricoles » 31800 Villeneuve-de-Rivière France

**Keywords:** Heterogeneity of infection, Endoparasites, Parasite distribution, Resources, Vegetation structure, Ungulate

## Abstract

Parasitic infection by endoparasites is heterogeneous within a population. Such heterogeneity in parasitic status among individuals depends in particular on differences in their susceptibility to infection and in the habitats and resources used by the individuals. While several studies have aimed to identify individual factors and, mostly at large spatial scales, environmental factors that influence endoparasitism in wild populations, we aim in this study to investigate the influence of habitat quality (vegetation type, resource availability) on parasite burden within a population of roe deer living in a heterogeneous forest. We collected 1,469 fecal samples to measure the parasite burden on 952 roe deer captured between 1996 and 2020 in Chizé (France), a study site stratified into two contrasting sectors in terms of vegetation structure and resource quality. We quantified the effect of the sector on parasitism after considering the possible influences of age, sex, body mass and Julian date. The prevalence of parasitism was higher in individuals living in the poorer sector, but the intensity of the parasite burden was not influenced by the sector. These results suggest that within a host population, parasite infection risk would not be the same everywhere, probably due to differences in resource availability, vegetation species and density of host, showing the need to study parasitism at fine scales.

## Abstract (fr)

L’infection parasitaire par les endoparasites est hétérogène au sein d’une population. Cette hétérogénéité du statut parasitaire entre individus dépend notamment des différences de sensibilité à l’infection et des habitats et ressources utilisés par les individus. Alors que plusieurs études ont cherché à identifier les facteurs individuels et, principalement à grande échelle spatiale, les facteurs environnementaux influençant l’endoparasitisme dans les populations sauvages, nous souhaitons, dans cette étude, étudier l’influence de la qualité de l’habitat (type de végétation, disponibilité des ressources) sur la charge parasitaire au sein d’une population de chevreuils vivant dans une forêt hétérogène. Nous avons collecté 1 469 échantillons fécaux afin de mesurer la charge parasitaire sur 952 chevreuils capturés entre 1996 et 2020 à Chizé (France), un site d’étude stratifié en deux secteurs contrastés en termes de structure de la végétation et de qualité des ressources. Nous avons quantifié l’effet du secteur sur le parasitisme après avoir pris en compte les influences possibles de l’âge, du sexe, de la masse corporelle et de la date julienne. La prévalence du parasitisme était plus élevée chez les individus vivant dans le secteur le plus pauvre, mais l’intensité de la charge parasitaire n’était pas influencée par le secteur. Ces résultats suggèrent qu’au sein d’une population hôte, le risque d’infection parasitaire ne serait pas le même partout, probablement en raison de différences de disponibilité des ressources, d’espèces végétales et de densité d’hôtes, ce qui souligne la nécessité d’étudier le parasitisme à des échelles fines.

## Introduction

Studying the ecology of endoparasite populations is crucial to better understand and predict their impact on their hosts. In wild populations, multiple factors drive endoparasite circulation, including climatic and environmental influences on the biology of the parasite, as well as the host itself [3, 33]. With their complex life cycle (*i.e.*, several hosts, passage in the environment between each host), the population dynamics of endoparasites are influenced by environmental conditions (*e.g.*, population density and climatic effects) [14, 45], but also by the individual characteristics of their host (*e.g.*, immune status, body condition and behavior) [10, 13, 30]. Endoparasite burdens are often studied at large spatial scales in wild populations [28, 32], for example by comparing different populations living in different habitats (*e.g.*, [13, 24]), rather than testing for a potential spatial structure of the parasite burden within a population. Yet, at this scale, the structure of the infection can depend on several factors, *e.g.*, on local environmental and climatic conditions and host populations [4, 31]. For example, Albery *et al.* [2] showed that the intensity of *Fasciola hepatica* in a red deer population seemed to be strongly influenced by the presence of wet grazing areas on a site of approximately 12 km^2^. It is therefore important to better understand the spatial patterns of infection, considering different scales.

One of the key factors that can influence fine-scale endoparasitism is the host itself, including its behavior and movements in the environment to acquire resources (*i.e.*, dependent on the availability of food resources, their quality and competition for them) [26], but also its ability to eliminate a parasite. Indeed, when the quantity or quality of food resources are insufficient, the physical condition of individuals can be negatively impacted, leading to a decrease in their ability to control parasites [23]. Conversely, increased parasitism can occur when local host density is high or food resources are rare, implying that the hosts forage in the same location, increasing risk of parasite transmission in this area [19], as for several endoparasite species, their life cycle includes a free-living phase in the environment (*e.g.*, trichostrongylids) [34]. Hence, the infection rate of a host may depend on its feeding behaviour and on its previous nutritional status, but also on the quality of the diet during the period of infection [43]. Therefore, within a population, individuals living in different types of habitat can have very different immune phenotypes and parasitic burden (*i.e.*, sheep [33]; red deer [2]). Furthermore, the relationship between body condition and parasitic infections can be bidirectional: individuals with lower body condition may be more susceptible to parasitism due to weakened immune responses, while increased parasitism can lead to further deterioration in the host’s physical state [9]. Thus, individuals within a population, living in different habitats or with varying body conditions, may exhibit diverse immune phenotypes and parasitic burdens, as demonstrated in species such as sheep [30] and red deer [2].

At a fine scale, host parasite burden can also be influenced by the environmental conditions in which the parasites live, including for instance weather conditions and vegetation type. Climatic parameters such as temperature, humidity or exposure to UV rays can considerably influence the survival of parasites [22], but also their activity when they are in the free-living stage. For example, in digestive strongyles, rain or the amount of dew water are known to be decisive in ensuring the movement of larvae trapped inside the feces towards the grass [42]. Local meteorological conditions and microclimates, which can be influenced by vegetation, can directly influence the development, survival and dispersal of free-living stages of endoparasites (*e.g.*, for gastro-intestinal strongyles [34]) or their intermediate hosts (*e.g.*, snails and slugs for protostrongylids). Parasites develop and survive better in microhabitats with humidity and low exposure to excessively extreme climatic conditions (*e.g.*, high heat or drought). However, although the influence of weather conditions on endoparasites is well documented, studies on the influence of vegetation cover on endoparasitism are scarce in the literature, particularly in forest environments and at fine scale. To our knowledge, no studies have investigated the influence of forest structure and the quality of resources for hosts on their endoparasite burden in a forested environment.

We aimed in this study to investigate the influence of forest structure as a spatial determinant of the parasite burden within a host population living in a habitat with variable environmental conditions. Using fecal samples, we measured parasite burden of four groups of parasites (*i.e.*, gastro-intestinal strongyles, *Trichuris* sp., *Eimeria* spp. and protostrongylids) in a roe deer (*Capreolus capreolus*) population living in Chizé. This study site in France has two sectors of contrasting habitat quality [38], *i.e.*, variable quantity and quality of resources. Previous studies in Chizé have also shown that individuals living in the richest part of the study site have higher body mass [37, 38], reflecting spatial heterogeneity in body condition of roe deer. We can therefore expect that the endoparasite burden of roe deer would differ depending on forest cover and predicted that this burden, in terms of prevalence and intensity, would be higher in individuals facing poor environmental conditions such as low availability of resources, compared to individuals living in richer environments.

## Material and methods

### Ethics approval

This research was conducted with the approval of the French authorities (French Ministry of Environment) and performed in accordance with the conditions detailed in the specific accreditation issued to the “Office Français de la Biodiversité” by the “Préfecture de Paris” (Agreement No. 2009–014, No. 2013–118, and No. 2019-02-19-003). The experiments were carried out by respecting the European and French laws defined for the ethical use of animals in research.

### Study site and habitat quality

**Figure 1:**
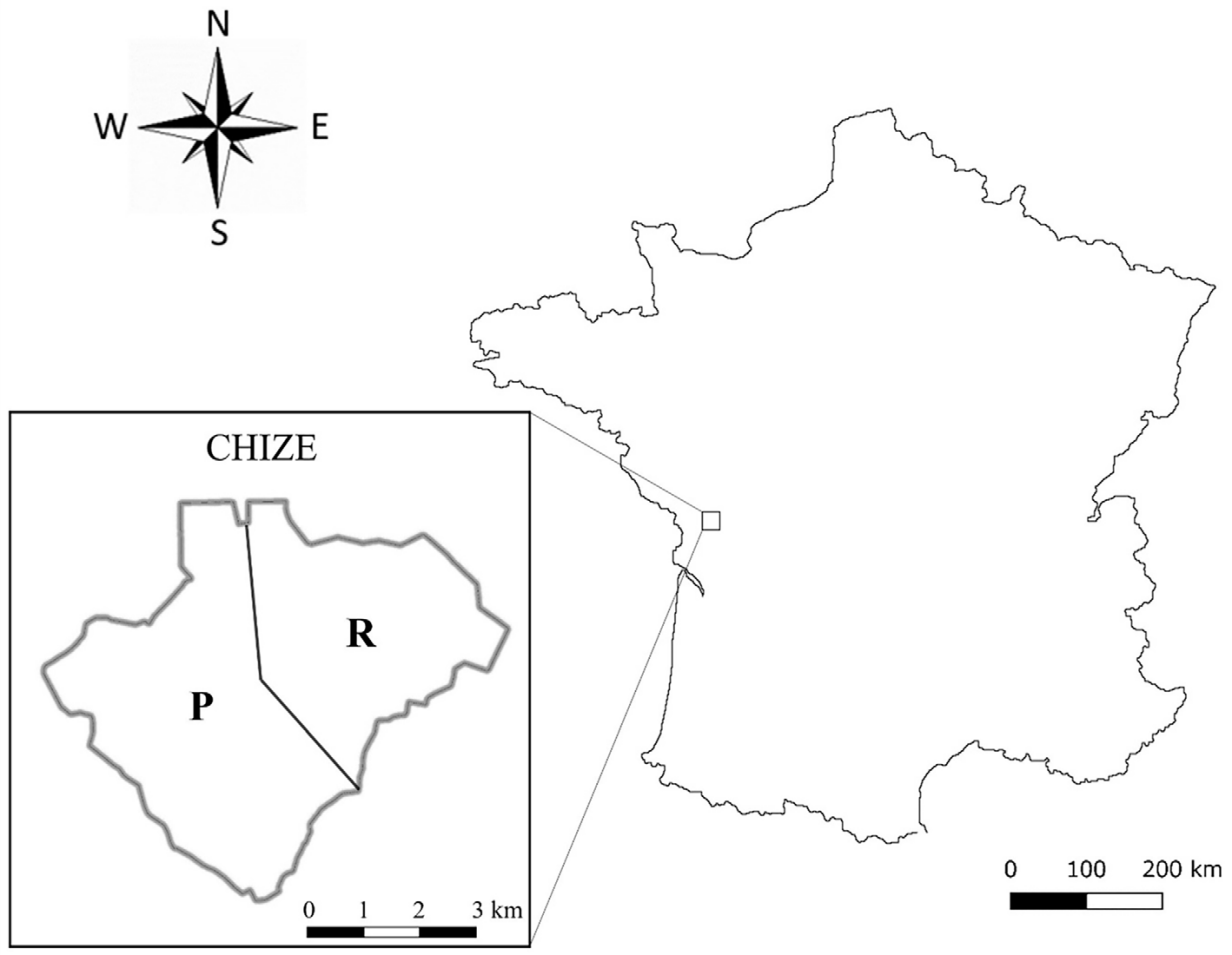
Maps of the study site of Chizé stratified into two habitats with its location in France. On the left: the two sectors characterized as richer (R) and poorer (P). This figure was carried out using Qgis 3.16 software.

This work was conducted on the population of roe deer living in the “Réserve Biologique Intégrale of Chizé”, an enclosed forest of 2,614 ha managed by the “Office National des Forêts”. This study site is located in western France (46° 50’ N, 0° 25’ E) (Fig. 1), where the climate is oceanic with mild winters and hot, dry summers.

The forest of Chizé presents low productivity due to poor quality of the soil and the frequent summer droughts. Moreover, based on a geological map [29], the distribution of the main plant/tree species and the nitrogen content of the plants [37], we determined two habitats of contrasting quality within the reserve [38]. This spatial pattern at this study site is an opposition between the north-eastern area and the rest of the reserve (north-western and southern areas) (Fig. 1). The north-eastern area, composed by oak (*Quercus* sp.) and hornbeam (*Carpinus betulus*) as the dominant species associated with hawthorn (*Crataegus monogyna*), dogwood (*Cornus* spp.), and maple (*Acer* sp.) (many herbaceous plants, mainly and/or preferred food eaten by the roe deer during spring or summer) [18], is the richest area in terms of resources, also reflected by a greater mass of fawns compared to the other part of reserve [36]. The other part of the study site is considered poorer and is stratified into two habitat types: the north-western part which is represented by oak (*Quercus* sp.), maple (*Acer* sp.) with butcher’s broom (*Ruscus aculeatus*), and the south part which mainly contains beech (*Fagus sylvatica*) and which is the poorest habitat in Chizé. Thus, for the following analyses, we will use the term “sector” to define these two types of habitat at Chizé: “sector R” for the north-eastern area (richer area), “sector P” for the north-western and southern areas (poorer area) (Fig. 1).

### Data collection

Data on roe deer were collected during a Capture-Mark-Recapture program. Since 1977, roe deer captures by drive-netting have taken place every winter (January–March) in Chizé, with 10–12 capture days per year (see [21] for further details). Once animals were captured, experiments were performed between 1 and 4 h and information was recorded or biological samples collected, *e.g.*, age, sex, body mass (in kg, to the nearest 0.05 g) and fecal matter (used to measure parasite burden). In this study, we used data only from individuals of known age (*i.e.*, captured within their first year of life) in the period from 1996 to 2020, except for the year 2000 (no captures carried out after the hurricane “Lothar” of 1999) [21] and the years from 2003 to 2007 (no data collected on parasitism in roe deer).

**Figure S1:**
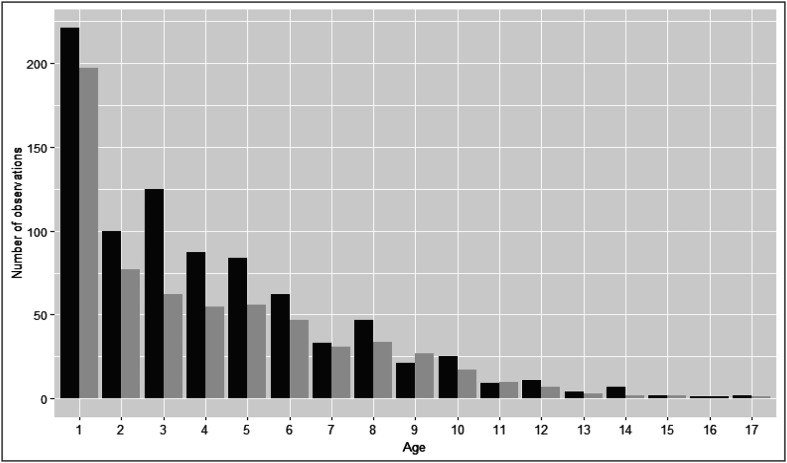
Number of observations according the age of roe deer and the sector. Black, sector R; light grey, sector P.

The total dataset consisted of 1,469 measurements from 952 individuals of known age (*n* = 418, 819 and 232 measurements on 1, 2–7 and 8–17-years old individuals, respectively). Among the 952 roe deer studied, 655 were captured once, 165 twice, 77 three times, 31 four times, and 24 five to seven times. Furthermore, these numbers are indicative of the total dataset, but the number of data used to make the analyses was dependent on the measure of parasitism used. Approximately 60% of roe deer were captured in the R sector and 40% in the P sector. For roe deer close to the boundary of the two sectors, we assigned the sector most used by the animal based on accurate telemetry monitoring in the field. Each age was represented in each sector, except an individual of 16 years in sector P (see the distribution of data by age and by sector on Figure S1). The sex-ratio in each sector was approximately balanced (sector R: 51% females, 49% males; sector P: 54% females, 46% males). Moreover, roe deer with on average higher body condition were in sector R [19.67 ± 4.46 kg] rather than in the sector P [18.97 ± 4.85 kg] (Mann & Whitney, W = 357029, *p*-value = 0.005).

### Measures of parasite burden

At Chizé, in roe deer, we had several categories of eggs or larvae identifiable during the analysis of feces: lung parasites, the protostrongylids (nematodes), and digestive tract parasites such as *Eimeria* spp. (coccidian: Protozoa) and nematodes, *i.e.*, gastrointestinal strongyles (GI strongyles, including several species belonging mostly to *Trichostrongylidae* family [8]) and *Trichuris* sp. We considered feces eggs/larval count as a marker of positivity to the parasite (*i.e.*, classic estimation in domestic ruminants to assess parasitic infections). Fecal samples were analysed with the Baermann technique [6] to isolate and count the first stage larvae of pulmonary nematodes in larvae per gram of feces (lpg). We also performed a modified McMaster protocol [40] with a flotation solution of magnesium sulfate (MgSO_4_, s.g. = 1.26; 1996–2013) or zinc sulfate (ZnSO_4_, s.g. = 1.36; 2014–2020) to count the number of eggs/oocysts per gram of feces (epg/opg for digestive parasites, *i.e.*, helminths and coccidian). Briefly, for each sample, we mixed 2.5–5 g of feces with the flotation solution (1:15 dilution ratio). We then homogenized the solution and sampled 1 mL that was loaded on a McMaster slide with two chambers. We thus counted the number of parasite propagules in the whole chambers (quantitative examination; theoretical sensitivity of 15 epg/opg). We also centrifuged a 14 mL tube with the remaining solution, covered with a coverslip. We thus placed the coverslip on a microscope slide before searching with a microscope (lens: ×10) for the presence of parasite propagules (“control slide”; qualitative examination). We attributed the value of 7.5 epg/opg (rounded up to the nearest whole number for statistical analyses) to parasites with no eggs/oocyst observed on the McMaster slide, but at least one egg/oocyst observed on the control slide.

### Statistical analyses

#### Response variables

To assess parasite infections in roe deer according to different factors, we used two measures of the level of parasitism in a host as response variables: a) parasite prevalence assessed by the individual presence/absence of parasites propagules; b) parasite intensity estimated by the number of fecal parasite propagules in infested individuals only. We only considered the most prevalent parasites in the population of Chizé: GI strongyles, *Trichuris* sp, *Eimeria* spp. and protostrongylids. We ran separate analyses for each parasite and measure of the level of parasitism. We analyzed prevalence using a generalized linear mixed model (GLMM) with a binomial error. For the intensity analysis, parasite egg count distributions are non-normal and strongly overdispersed. Just like the Poisson model, the negative binomial model is commonly utilized as a distribution to model such count data; it allows a variance higher than its mean. Using the “descdist” function from the “fitdistrplus” package [16], we confirmed that GI strongyles, *Trichuris* sp. and *Eimeria* spp. intensities followed a negative binomial distribution. However, it did not fit for protostrongylids intensities. This variable was thus log-transformed as log_10_(*x* + 1) to obtain a normal distribution and analyzed using linear mixed models (LMM). Moreover, the intensity of *Eimeria* spp. had about ten extreme values above 4,000 opg (*e.g.*, 210,000 opg); we therefore rounded all these values to 4,000 opg to improve the distribution.

#### Explanatory variables

We included in the models all the variables known to possibly affect parasite burden in roe deer: age, sex, body mass and Julian date. We accounted for possible effects of age (linear or quadratic effect, determined by model selection and in accordance with the publication of [13]) and sex because previous work has shown that in roe deer, juveniles and males are more infested than adults and females [10]. Moreover, roe deer with lower body mass are more parasitized than individuals with higher body mass [7, 10, 13], thus we considered this variable in our model. Body mass measured in young individuals (*i.e.*, first year of life) depends on the date of capture (gain on average 12 g/day during the capture period) [17] and on the sector in Chizé (up to 2 kg difference in the mass of fawns between sector R and sector P in Chizé) [36]. Therefore, we standardized juvenile body mass with Julian date and sector by taking residuals of a linear model. For adult roe deer (*i.e.*, ≥ 2 years old), body mass depends on the sex (males are heavier than females, Mann & Whitney test: *n* = 1162, W = 11986, *p*-value < 0.001), on the age (body mass does not change between the ages of 4 and 10 years in both males and female [38] but we need to control for 2, 3 and after 10 years old) and on the sector. We thus standardized the adult body mass by these parameters (linear effect of age and categorical effect of sector) by taking residuals of a linear model. The Julian date of capture (Day 1 = January 1st) was also added to control for potential among-individual differences in parasite burden and body mass due to the timing of sampling. The median day of sampling was day 37 of the year corresponding to February 7th [January 7th; March 13th] 95% CI.

To avoid pseudo-replication problems [25], we included the factors individual identity (*i.e.*, individuals can be captured between 1 and 10 times in our dataset) and year of capture as random effects. This allowed us to control for unexplained variance due to among-individual and inter-annual variations (including the potential influence of changing the flotation solution – MgSO_4_ and ZnSO_4_ – to measure parasitism during the study period).

To test our prediction, we performed model selection for each group of parasites and parasitism measure (intensity and prevalence) by fitting a null model (*measure of parasite burden ~ age + sex + body mass + Julian date + 1|year of capture + 1|individual identity*) including all the previously described variables and we compared this model (“null model”) to a model (“sector model”) also including the sector of capture (two categories: R and P), as a marker of local resources quality and quantity (*i.e.*, habitat quality).

#### Model selection

Based on the “corrected” Akaike’s information criteria (AICc) [12], we selected the best model describing variation of each parasite prevalence/intensity by comparing complete and null models (Table 1). For each response variable, we retained the model with the lowest AICc. When competing models differed in AICc (ΔAICc) by less than two, we retained the most parsimonious one, *i.e.*, the model with fewer parameters. Finally, we assessed the goodness of fit by calculating the marginal (R2m; variance explained only by fixed effects) and conditional (R2c; variance explained by the entire model) variances using the r.squaredGLMM function of the MuMIN package (Bartoń 2019). For each model selected, the normality of the residuals was evaluated visually.

**Table 1:** Prevalence (% presence) and intensity of: GI strongyles (egg/g of feces), *Trichuris* sp. (egg/g of feces), *Eimeria* spp. (oocyst/g of feces), protostrongylids (larvae/g of feces) according to the sector of Chizé as well as the total at the site.

	Prevalence			Intensity			
	Sector	*n*	%	*n*	Mean ± SD	Min	Max
GI strongyles	R	841	54	451	62.31 ± 110.26	8	1,050
	P	628	60	375	63.89 ± 121.86	8	1,440
*Trichuris* sp.	R	837	53	442	317.61 ± 918.05	8	9,500
	P	629	58	367	295.08 ± 663.99	8	6,750
*Eimeria* spp.	R	838	34	283	1,114.12 ± 12,568.91	8	210,200
	P	629	40	253	1,194.81 ± 11,699.25	8	180,000
protostrongylids	R	801	21	168	23.83 ± 91.44	0.16	900
	P	621	25	157	38.48 ± 114.33	0.09	766.8

All statistical analyses were performed using R (version 4.2.2) [39]. We fitted models using the functions lmer, glmer and glmernb from package lme4. Significance threshold was set at the *α* = 0.05 level. Maps were performed using Qgis 3.16 software.

## Results

### Data description

The most prevalent parasites in roe deer in Chizé were GI strongyles and *Trichuris* sp., observed in more than half of the individuals (56% and 55%, respectively), while *Eimeria* spp. and protostrongylids were less prevalent (37% and 23%, respectively). In terms of parasite intensity, the mean number of eggs/oocysts/larvae per gram of feces ± standard deviation was: 63.0 ± 115.6 epg for GI strongyles (*n* = 1,469), 307.4 ± 812.3 epg for *Trichuris* sp. (*n* = 1,466), 1152.6 ± 12138.5 opg for *Eimeria* spp. (*n* = 1,467) and 30.9 ± 103.2 lpg for protostrongylids (*n* = 1,422).

### Parasitic prevalence

Globally, a higher parasite prevalence was observed in roe deer living in sector P (Table 1). Based on model selection, parasitic prevalence (GI strongyles, *Trichuris* sp. and *Eimeria* spp.) was best described by the models including the sector effect (ΔAICc > 2; Table 2), except for the protostrongylids (ΔAICc < 2; Table 2). However, for this parasite, the two models had a very close AICc (ΔAICc = 0.5).

**Table 2:** Performance of linear and generalized linear mixed models (sector and null models) to explain the variation in the prevalence and intensity of each group of parasites according to different predictors in fixed effect (age, sex, body mass, Julian date and sector) and in random effect (year of capture and individual identity). The best model (in bold) is the model reflecting the best compromise between accuracy and complexity of the model. ΔAICc is the difference in AICc between the two models.

Response variable		Model	AICc	ΔAICc
Prevalence	GI strongyles	***Sector***	1,823.1	0
		*Null*	1,827.7	4.6
	*Trichuris* sp.	***Sector***	1,683.5	0
		*Null*	1,686.7	3.2
	*Eimeria* spp.	***Sector***	1,684.9	0
		*Null*	1,688.8	3.9
	protostrongylids	***Null***	1,090.2	0
		*Sector*	1,090.7	0.5
Intensity	GI strongyles	***Null***	7,969.3	0
		*Sector*	7,970.6	1.3
	*Trichuris* sp.	***Sector***	9,779.2	0
		*Null*	9,779.4	0.2
	*Eimeria* spp.	***Null***	6,676.0	0
		*Sector*	6,677.1	1.1
	protostrongylids	***Null***	710.9	0
		*Sector*	715.9	5.0

As expected, a negative effect of body mass on prevalence was found for each parasite (Table 3), meaning that individuals with lower mass were more parasitized overall. Age was also a factor structuring the prevalence of GI strongyles, *Eimeria* spp. and protostrongylids (Table 3), with young roe deer (1 year old) being more often parasitized compared to adults and old individuals (see Figure S2). Julian date also had a significant effect on the prevalence of *Trichuris* sp. and *Eimeria* spp. with a higher prevalence later in the catching season. Finally, only the prevalence of *Trichuris* sp. was sex-specific, with males being more parasitized than females.

**Table 3:** Best model set descriptions and extracted values for the prevalence of each parasite species. In bold: significant effect (*p*-value < 0.05) of the predictor in each model.

Parasite prevalence (Response variable)	Best model	Significant parameters in the model selected	Estimate ± SE	*z*-value	*p*-value	R^2^m	R^2^c
GI strongyles (*n* = 1469)	*Sector*	Intercept	0.12 ± 0.14	0.85	0.39	0.11	0.20
		Age	−0.17 ± 0.06	−2.70	**0.007**		
		Sex (Male)	0.18 ± 0.13	1.42	0.15		
		Body mass	−0.67 ± 0.07	−9.09	**<0.001**		
		Julian date	0.01 ± 0.07	0.20	0.84		
		Sector (P)	0.33 ± 0.13	2.56	**0.01**		
*Trichuris* sp. (*n* = 1466)	*Sector*	Intercept	−0.01 ± 0.17	−0.08	0.93	0.22	0.40
		Age	−0.02 ± 0.07	−0.21	0.84		
		Sex (Male)	0.41 ± 0.16	2.64	**0.01**		
		Body mass	−1.18 ± 0.11	−11.24	**<0.001**		
		Julian date	0.16 ± 0.08	2.02	**0.04**		
		Sector (P)	0.35 ± 0.15	2.27	**0.02**		
*Eimeria* spp. (*n* = 1467)	*Sector*	Intercept	−0.75 ± 0.12	−6.44	**<0.001**	0.17	0.23
		Age	−0.56 ± 0.07	−7.72	**<0.001**		
		Sex (Male)	−0.15 ± 0.13	−1.12	0.26		
		Body mass	−0.70 ± 0.07	−9.34	**<0.001**		
		Julian date	0.14 ± 0.07	2.12	**0.03**		
		Sector (P)	0.32 ± 0.13	2.44	**0.01**		
Protostrongylids (*n* = 1422)	*Null*	Intercept	−2.69 ± 0.39	−6.86	**<0.001**	0.33	0.51
		Age	−0.67 ± 0.16	−4.13	**<0.001**		
		Age^2^	0.32 ± 0.09	3.41	**<0.001**		
		Sex (Male)	0.23 ± 0.21	1.09	0.28		
		Body mass	−1.59 ± 0.20	−8.07	**<0.001**		
		Julian date	0.001 ± 0.006	0.14	0.89		

### Parasitic intensity

Parasitic intensity for protostrongylids tended to be higher in roe deer in sector P than in sector R, while for GI strongyles and *Eimeria* spp. the difference was low, and for *Trichuris* sp., roe deer in sector P tended to have lower average intensity of infection than in sector R (Table 1).

Based on model selection, parasitic intensity was best described by the null models (*i.e.*, without the sector effect) for three groups of parasites, except *Trichuris* sp. (Table 1). For GI strongyles and *Eimeria* spp., the models including the sector effect had close AICc (ΔAICc = 1.3 and 1.1, respectively) with the null models. For *Trichuris* sp., the intensity was higher in sector P, but not significantly (*p* = 0.13). As for models selected on prevalence, a negative effect of body mass on the intensity was found for each parasite (Table 4), meaning that individuals with lower body mass had overall higher intensities of parasites. Age was also a factor structuring the intensities of all parasites except GI strongyles (Table 4), with young roe deer (1 year old) having higher intensities of parasites than adults and older individuals (2–17 years old). Julian date and sex had no significant effects on parasitic intensities.

**Table 4:** Best model set descriptions and extracted values for the intensity of each parasite species. In bold: significant effect (*p*-value < 0.05) of the predictor in each model.

Parasite intensity (response variable)	Best model	Significant parameters in the model selected	Estimate ± SE	*z*- or *t*-value	*p*-value
GI strongyles (*n* = 826)	*Null*	Intercept	3.62 ± 0.14	25.04	**<0.001**
		Age	0.07 ± 0.03	2.11	**0.04**
		Sex (Male)	0.08 ± 0.07	1.22	0.22
		Body mass	−0.36 ± 0.03	−10.55	**<0.001**
		Julian date	−0.04 ± 0.03	−1.04	0.30
*Trichuris* sp. (*n* = 809)	*Sector*	Intercept	4.43 ± 0.20	21.83	**<0.001**
		Age	−0.14 ± 0.05	−2.91	**0.004**
		Sex (Male)	0.19 ± 0.10	1.90	0.06
		Body mass	−0.77 ± 0.05	−16.13	**<0.001**
		Julian date	−0.001 ± 0.05	−0.02	0.98
		Sector (P)	0.15 ± 0.10	1.51	0.13
*Eimeria* spp. (*n* = 536)	*Null*	Intercept	4.60 ± 0.16	28.56	**<0.001**
		Age	−0.27 ± 0.07	−3.72	**<0.001**
		Sex (Male)	0.04 ± 0.14	0.30	0.76
		Body mass	−0.44 ± 0.07	−6.12	**<0.001**
		Julian date	−0.07 ± 0.07	−0.95	0.34
Protostrongylids (*n* = 325)	*Null*	Intercept	0.59 ± 0.09	6.42	**<0.001**
		Age	−0.09 ± 0.04	−2.12	**0.03**
		Sex (Male)	−0.05 ± 0.08	−0.67	0.51
		Body mass	−0.29 ± 0.04	−7.19	**<0.001**
		Julian date	0.07 ± 0.04	1.81	0.07

## Discussion

Understanding the spatial pattern of parasite burden in large mammals often depends on environmental conditions, such as the habitat and climatic/micro-climatic conditions where hosts and parasites are found. This study aimed to assess whether differences in forest structure and sector-based habitat quality influenced parasite burden in roe deer within the Chizé study site. We provided evidence that the level of parasitism varied among the two sectors with contrasted habitat quality in Chizé, after considering possible confounding effects of phenotypic attributes. Interestingly, we observed a higher prevalence of parasites in individuals living in the sector with poor habitat quality compared to high-quality habitat. However, our results do not seem to show an effect of sector on parasite intensity.

First, considering effects of phenotypic attributes in our models, we found mainly the effects of body condition and age on parasite burden. Higher parasite prevalence (GI strongyles, *Eimeria* spp., protostrongylids) and higher parasite intensity (*Trichuris* sp., *Eimeria* spp., protostrongylids) were found in younger roe deer, which is generally in agreement with the results shown in the studies of Body *et al.* [10] and Cheynel *et al.* [13]. Body condition was also a factor that emerged in all our models, showing that roe deer in poorer body condition were more parasitized by all parasites studied. This result is consistent with other studies on roe deer parasitism [10, 13]. Regarding sex, this factor only emerged once in our models, with a higher prevalence of *Trichuris* sp. in males. At Chizé, it is therefore assumed that sex does not appear to be a dominant factor driving parasite burden, probably because other determinants of parasitism are more important, such as age or body condition, or environment and habitat sector.

Our results showed that roe deer were more often parasitized in sector P (Table 2). This could be explained by the lower immune capacities of the roe deer against parasites in this sector compared to the individuals in sector R. An individual needs energy to ensure various physiological functions, such as growth and reproduction, which prevents the exclusive allocation of resources to immune defenses [46]. The quality and availability of food resources strongly determine the allocation of energy to immune defence, with defences being improved when the resources are richer and more abundant [27, 35]. In Chizé, with contrasted quality of habitat (quality or quantity of resources) in the two sectors, heavier roe deer were found in sector R (19.67 ± 4.46 kg) than in sector P (18.97 ± 4.85 kg). In addition to oaks, the richer sector is composed of hornbeam, hawthorn and dogwood, associated with herbaceous species such as bluebells (*Hyacinthoides nonscripta* L.) or Pyrenees star of Bethlehem (*Ornithogalum pyrenaicum* L.), which are highly appreciated by roe deer [18, 37]. These plants are more frequent in this sector and are known to have a high nitrogen content [37], making sector R a habitat with generally richer resources for roe deer. This could explain why roe deer in sector P have higher parasite prevalence. In addition, parasites can reduce the host’s body condition by spoliating resources and reducing its appetite. Parasites will thus negatively influence the body condition of their host, proportionally to the level of infestation. This could explain the negative covariance between body mass and parasitism we observed here and in previous studies, *e.g.*, [5, 10, 11, 44]. As a result, hosts will be even less able to defend themselves, which could lead in higher parasitic infestation and further deterioration of its body condition and a “vicious circle” is created [9]. A study has, for example, shown that the presence of parasites increases the reduction of trace elements such as copper, iron or even zinc, essential for intestinal immunity, and thus influence the expression of disease [1, 15]. Therefore, parasitized individuals are more likely to be negatively affected if they do not have the energy resources necessary to defend themselves.

The higher parasite prevalence in sector P (Table 1) could also come from a higher rate of encounters with parasites in the environment or with infested hosts, due to the structure/composition of the forest and roe deer behavior and density. As mentioned above, sector R is mainly composed of oaks, hornbeams, hawthorns, dogwoods and herbaceous species, while sector P contains oaks but also other forest species such as maple or beech, and in particular many more butcher’s broom (*Ruscus aculeatus*). This denser vegetation creates a more closed environment in sector P [36], particularly due to the extensive presence of butcher’s broom (pers. obs.), which may create microhabitats with higher humidity. Such conditions are known to promote the development, migration and survival of free-living stages of some parasites (*e.g.*, strongyles [34]) or intermediate hosts (*e.g.*, snails and slugs for Protostrongylids) that require moist environments for development. Although few studies have directly assessed the influence of vegetation structure and microclimate on parasite transmission in roe deer, Body *et al.* [10] reported a positive relationship between summer rainfall and gastro-intestinal strongyles intensity in female roe deer, supporting the idea that moisture-related factors can influence infection dynamics in this species. However, groups and species of parasites have different sensitivity to environmental conditions, and heterogeneity in habitat and microclimate can be high within each sector. Refining identification of parasites (*e.g.*, identification of the different parasite species rather than considering groups of parasites such as GI strongyles) and the vegetation structure (cover and vegetation species) could help to better understand the dynamic of infection in the different sectors. Regarding the behavior of roe deer, given that sector P is a poorer habitat, roe deer can move further to find resources. A study by Saïd *et al.* [41] has shown that female roe deer living in low quality areas adjusted the size of their home range to include more patches of habitat and compensate for the lack of resources. Thus, intraspecific competition for resources could be present in the same part of the sector. The roe deer in this sector may move further to find resources and therefore encounter more areas where other infected roe deer have defecated and where infesting larvae are present, increasing the risk of being infested.

Finally, the density of a host population is also known to influence their level of parasitism [10]. Unfortunately, we have no recent information on roe deer density in each sector, but we know that in a heterogeneous environment, individuals are expected to distribute in an ideal free manner, with higher density of individuals in the richer habitats, so that resources are partitioned equally among individuals [20]. Previous works have shown that there is a heterogeneity of habitat in Chizé and that roe deer follow this rule, with higher density of individuals in the richer sector [36, 37]. Local density could therefore not explain the higher prevalence of parasites in sector P. However, this could help us to understand the lack of differences in parasitic infestation between the two sectors, with higher environmental parasitic pressure associated with higher density in sector R, counterbalancing higher sensitivity to infection associated with low resource quality and quantity in sector P.

## Conclusion

To conclude, the results of this study are consistent with other works on the subject, showing that parasitism can be dependent on quality of habitat [2, 31, 33], even in a predominantly forested environment. The spatial trend seen (mainly in prevalence) demonstrates that higher or lower parasitism can be associated with different sectors of a given study system, signifying that uneven sampling in space could introduce confounding variation and bias. It is, however, difficult to understand the influence of host density, habitat and resources on the abundance of endoparasites, in particular because it is difficult to disentangle their direct effect on parasite replication from their indirect effect on pathogen mortality, mediated by the immune system. Furthermore, while seasonal variation and the longevity of infective parasite stages in the environment could play a significant role in parasite dynamics, our study was, unfortunately, limited by the lack of data on these environmental stages and the duration of infection in individual roe deer. As such, our findings represent a snapshot of infection status at the time of sampling, rather than reflecting long-term infection dynamics. Future studies should integrate different aspects of host density, and immune, physiological and behavioral responses at even finer spatial scales to better identify the mechanisms of parasite transmission and maintenance in a host population. At the same time, it would be interesting to study the vegetation species and diet (quality: N, P, C and minerals) of the host population according to habitat structure, but also to define the local density according to sector, as the density of the host population can have an impact on parasite infection in a population.

## Figures and Tables

**Figure S3:**
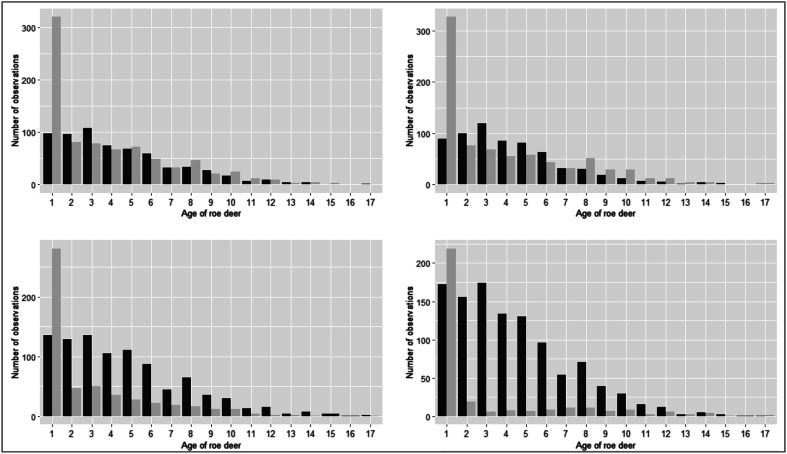
Presence/absence of a) Gastrointestinal strongyles, b) *Trichuris* sp., c) *Eimeria* spp. and d) Protostrongylids in roe deer according to the age of individuals. Black, absence of parasites; Light grey, presence of parasites.

## Supplementary material

The supplementary material for this article can be found at https://www.parasite-journal.org/10.1051/parasite/2025041/olm.
